# Regulation of MAVS Expression and Signaling Function in the Antiviral Innate Immune Response

**DOI:** 10.3389/fimmu.2020.01030

**Published:** 2020-05-27

**Authors:** Zhihua Ren, Ting Ding, Zhicai Zuo, Zhiwen Xu, Junliang Deng, Zhanyong Wei

**Affiliations:** ^1^Key Laboratory of Animal Disease and Human Health of Sichuan Province, College of Veterinary Medicine, Sichuan Agricultural University, Chengdu, China; ^2^The College of Animal Science and Veterinary Medicine, Henan Agricultural University, Zhengzhou, China

**Keywords:** innate immunity, MAVS, molecular regulation, viral replication, immune evasion

## Abstract

Viral infection is controlled by host innate immune cells that express specialized receptors for viral components. Engagement of these pattern recognition receptors triggers a series of signaling pathways that culminate in the production of antiviral mediators such as type I interferons. Mitochondrial antiviral-signaling protein (MAVS) acts as a central hub for signal transduction initiated by RIG-I-like receptors, which predominantly recognize viral RNA. MAVS expression and function are regulated by both post-transcriptional and post-translational mechanisms, of which ubiquitination and phosphorylation play the most important roles in modulating MAVS function. Increasing evidence indicates that viruses can escape the host antiviral response by interfering at multiple points in the MAVS signaling pathways, thereby maintaining viral survival and replication. This review summarizes recent studies on the mechanisms by which MAVS expression and signaling are normally regulated and on the various strategies employed by viruses to antagonize MAVS activity, which may provide new insights into the design of novel antiviral agents.

## Introduction

The innate immune system is the first line of host defense against pathogens. Innate immune cells express specific pattern-recognition receptors that recognize microbial components known as pathogen-associated molecular patterns (e.g., viral nucleic acids and proteins) and activate intracellular signaling pathways leading to the antiviral response (1). The major pattern recognition receptors include Toll-like receptors (TLRs), which are located both intracellularly and on the cell membrane; RIG-I-like receptors (RLRs) and Nod-like receptors (NLRs), both located in the cytoplasm; and some cytoplasmic DNA receptors such as DAI, IFI16, DDX41, cGAS, and AIM2 (2–4). The cellular localization of invading viral components provides the possibility that the host utilizes cytoplasmic pattern-recognition receptors to respond to these stimuli. Among viral components, double-stranded RNA (dsRNA) and single-stranded RNA (ssRNA) are recognized by TLR3 and TLR7/8 in endolysosomes, respectively (5–7). RLRs bind dsDNA and trigger antiviral response (8). Viral ssRNA and dsRNA are recognized by NLRP3 (9), while NOD2 interacts with ssRNA and induces interferon (IFN) production (10). Intracellular dsDNA of viral origin is also recognized by cytoplasmic DAI, IFI16, DDX41, cGAS, and AIM2 (11–15).

RLRs have been extensively studied as sensors of cytoplasmic viral dsRNA and their importance in controlling RNA virus infection is now clear. The RLR family has three members: retinoic acid-inducible gene I (RIG-I), melanoma differentiation-associated gene 5 (MDA5), and laboratory of genetics and physiology protein 2 (LGP2). RIG-I and MDA5 are typical pattern recognition receptors, whereas LGP2 is considered to be a regulator of RIG-I- and MDA5-mediated signal transduction (16, 17). Upon viral recognition, RIG-I and MDA5 interact with the mitochondrial antiviral-signaling protein (MAVS, also known as IPS1, VISA, and CARDIF), which induces activation of the transcription factors interferon regulatory factors 3 and 7 (IRF3/7) and nuclear transcription factor-κB (NF-κB). This process ultimately leads to the expression of multiple proinflammatory factors and antiviral genes, such as IFN and IFN-stimulated genes (ISGs), which inhibit viral replication and transmission (18–21). Given that MAVS is the key adapter protein known to be required for defense against RLR-recognized RNA viruses, this review will specifically focus on the regulation of MAVS expression and signaling function and their manipulation by viruses.

## Structure and Function of MAVS

**Figure 1:**
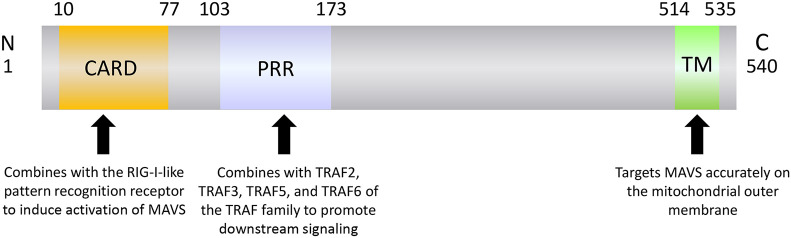
The structure of MAVS.

**Figure 2:**
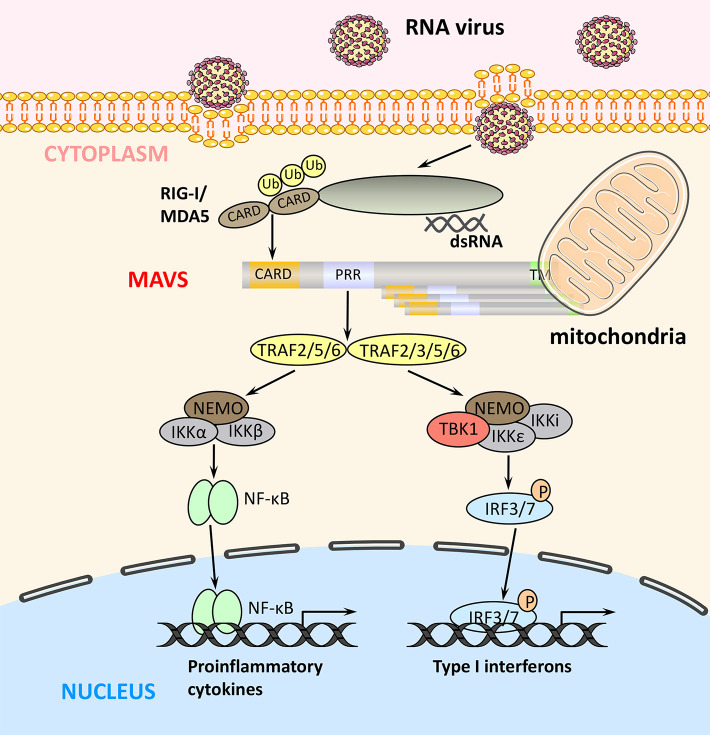
RLR signaling pathways. Binding of viral components to RIG-I and MDA5 induces their interaction with MAVS via their common CARDs. MAVS activates two signaling cascades leading to the production of immune factors. The TRAF2/5/6–IKK complex–NF-κB pathway induces transcription of proinflammatory cytokines, whereas the TRAF2/3/5/6–TBK1 complex–IRF3/7 pathway induces the expression of type I IFN genes.

MAVS is a 540-amino acid protein encoded by the nuclear genome (19). MAVS is mainly localized on the mitochondrial outer membrane, although it has also been detected on peroxisome and mitochondrial-associated endoplasmic reticulum membranes (22–24). MAVS contains three domains: an N-terminal caspase recruitment domain (CARD), a middle proline-rich region (PRR), and a C-terminal transmembrane (TM) domain (Figure 1). The MAVS CARD binds to similar CARDs present in RIG-I and MDA5, which induces MAVS activation (Figure 2). The MAVS PRR domain binds to the tumor necrosis factor receptor-related factor (TRAF) family members TRAF2, TRAF3, TRAF5, and TRAF6 to promote downstream signal transduction (25), and the TM domain ensures localization of MAVS to the mitochondrial outer membrane (20).

Upon binding of viral components, RIG-I and MDA5 undergo a conformational change that exposes their N-terminal tandem CARDs and forms the tetramer via CARDs (26), which are modified with K63-polyubiquitin chains by the E3 ubiquitin ligases TRIM25, RNF125, and RIPLET (27). In turn, K63-linked ubiquitination promotes binding of RIG-I and MDA5 to MAVS via their CARDs. The MAVS CARD rapidly forms prion-like aggregates, which convert other MAVS on the mitochondrial outer membrane into prion-like aggregates. This aggregation step is essential for the biological functions of MAVS (28). Next, MAVS binds through its PRR domain to TRAF2, TRAF3, TRAF5, or TRAF6, which promotes activation of TBK1 complex (containing TBK1, IκB kinase [IKK]i/ε, and NEMO) in the case of TRAF2/3/5/6 and of IKK complex (containing IKKα/β and NEMO) in the case of TRAF2/5/6 (Figure 2) (25, 29). The TBK1 complex promotes phosphorylation and homodimerization of IRF3 and/or IRF7, which then translocate to the nucleus where they bind to IFN-stimulated response elements and induce the transcription of target genes such as type I IFNs. Similarly, the TRAF2/5/6-activated IKK complex activates NF-κB to promote the transcription of proinflammatory cytokines (4, 8). Thus, the two MAVS-mediated signaling pathways play distinct but crucial roles in antiviral innate immunity (Figure 2).

## Regulation of MAVS Activity Under Physiological Conditions and During Viral Infection

The expression and function of MAVS are tightly regulated at the post-transcriptional and post-translational levels, which ensures that RLR signaling pathways are not only rapidly activated upon viral recognition but also curtailed in a timely manner upon viral clearance to avoid potentially harmful tissue damage.

### Post-transcriptional Regulation of MAVS

MAVS mRNA is polycistronic, and the key feature of polycistron is that protein translation can start not only from the N-terminal methionine, but also from the internal methionine, thus encoding for isoforms by alternative translation of distinct start sites (30, 31). MAVS mRNA was identified to produce a full-length and five CARD-deleted proteins (FL MAVS and MAVS-M142/303/358/367/449, respectively) from six distinct translational initiation sites based on methionine distribution. Under physiological conditions, binding of MAVS truncated isoforms to FL MAVS inhibits its spontaneous aggregation, while the formed aggregates are degraded by mitochondrial autophagy, thereby maintaining autoimmune homeostasis (31). In addition, MAVS-M142 cannot prevent the aggregation of FL MAVS during viral infection, but can block the production of IFN after MAVS-M142 binding to TRAF. However, the regulation of MAVS and MAVS-M142 can be achieved by an upstream open reading frame (uORF) in the 5′untranslated region (UTR). The translation of uORF might skip the start site of FL MAVS and initiate the translation of MAVS-M142 (32). Thus, the outcome of MAVS signaling is dictated to a certain extent by the net effects of FL MAVS and MAVS truncated isoforms. At present, only a few genetic studies have investigated the regulation of FL MAVS and MAVS truncated isoforms, and the mechanism underlying their functional competition is still unclear.

It is well-known that mRNA contains a number of regulatory elements in the 3′ and 5′ UTRs. Binding of regulatory factors such as proteins and complementary small RNA molecules to these elements plays an important role in controlling protein translation (33). For example, three AU-rich elements in the 3'UTR of MAVS mRNA mediates binding of human antigen R (HuR), which destabilizes the mRNA and maintains MAVS protein at low levels under normal physiological conditions (34). MicroRNAs (miRNA) are small non-coding RNAs that regulate gene expression by promoting the degradation and/or inhibiting the translation of target mRNAs (35). Four miR-27a-binding sites have been identified in the 3'UTR of MAVS mRNA, and up-regulation of miR-27a can inhibit MAVS expression (34).

The DEAD-box (DDX) family of highly conserved RNA helicases play important roles in RNA synthesis, processing, transport, and degradation (36). A recent study showed that, in virus-infected cells, DDX46 located in the nucleus binds to several conserved CCGGUU elements in MAVS, TRAF3, and TRAF6 mRNAs, which inhibits their translocation to the cytoplasm for translation and thus reduces MAVS, TRAF3, and TRAF6 protein levels (37).

### Post-translational Regulation of MAVS

**Figure 3:**
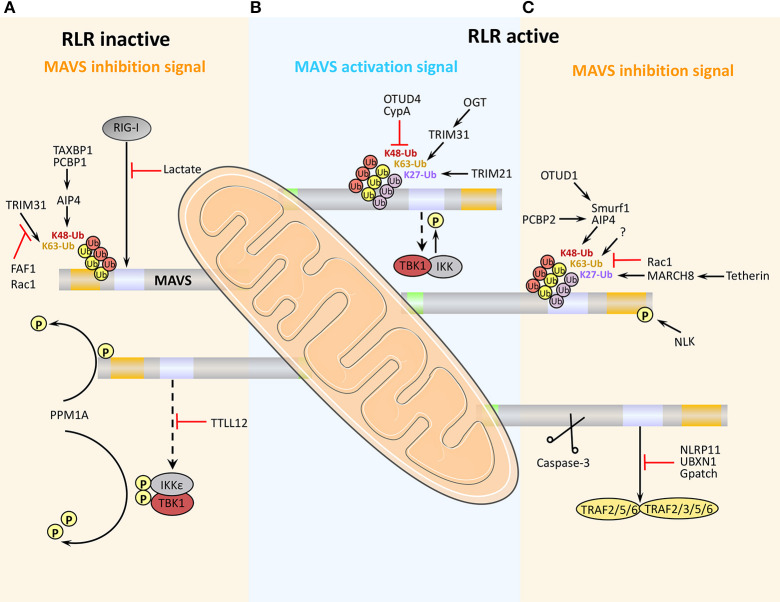
Positive and negative regulation of MAVS activity antiviral immunity. **(A)** Under physiological conditions, MAVS activity is inhibited to prevent aberrant activation of the immune response. **(B)** Early after viral infection, MAVS is activated by TRIM31-mediated K63-linked ubiquitination, and the inhibition of MAVS is relieved, thereby initiating the RLR signaling pathway. **(C)** At later stages of viral infection, MAVS is cleaved and degraded, which effectively arrests RLR-stimulated signaling.

MAVS can undergo a number of post-translational modifications (Table 1) that influence its function in promoting innate immune responses (Figure 3).

**Table 1:** The post-translational of modifications (PTMs) of MAVS.

**Regulator of**	**PTMs**	**Site**	**Regulatory effect**	**References**
**PTMs**				
?	K63-linked ubiquitination	K500	Promotes the recruitment of IKKε to MAVS, then IKKε detaches TRAF3 from mitochondria and promotes K63-linked deubiquitination of TRAF3 and its subsequent K48-linked ubiquitination degradation	(38)
TRIM31	K63-linked ubiquitination	K10/K311/K461	Promotes the aggregation and activation of MAVS during viral infection	(39)
OGT	Glycosylation	S366	Promotes the TRIM31-mediated K63-linked ubiquitination of MAVS and RLRs signaling	(40)
TRIM21	K27-linked ubiquitination	K325	Promotes TBK1 recruitment and thereby MAVS-mediated IFN production	(41)
MARCH8	K27-linked ubiquitination	K7	Promotes NDP52 to recognize K27-linked ubiquitination signal on MAVS and induces lysosomal autophagy of MAVS	(42)
TRIM25	K48-linked ubiquitination	K7/K10	Induces proteasomal degradation of MAVS	(43)
AIP4/ITCH	K48-linked ubiquitination	K371/K420	Induces proteasomal degradation of MAVS	(44)
PCBP1	K48-linked ubiquitination	NA	Promotes the K48-linked ubiquitination degradation of MAVS by recruiting AIP4 under normal conditions, thus preventing the spontaneous activation of immune response	(45)
PCBP2	K48-linked ubiquitination	NA	Promotes the K48-linked ubiquitination degradation of MAVS by recruiting AIP4 at the late stage of viral infection, thus avoiding excessive immune response	(44)
TAX1BP1	K48-linked ubiquitination	NA	Promotes the K48-linked ubiquitination degradation of MAVS by recruiting AIP4 under normal and viral infection conditions, thus playing a role similar to PCBP1/2	(46)
Smurf1 and Smurf2	K48-linked ubiquitination	NA	Induces proteasomal degradation of MAVS	(47, 48)
Ndfip1	K48-linked ubiquitination	NA	Promotes the K48-linked ubiquitination degradation of MAVS by recruiting Smurf1	(47)
OTUD1	K48-linked ubiquitination	NA	Promotes the K48-linked ubiquitination degradation of MAVS by recruiting Smurf1 at the early stage of viral infection, inhibiting the RLRs pathway	(49)
MARCH5	K48-linked ubiquitination	K7/K500	Induces proteasomal degradation of MAVS	(50)
RNF5	K48-linked ubiquitination	K362/K461	Induces proteasomal degradation of MAVS	(51)
OTUD4	Removal of K48-linked ubiquitination	NA	Stabilizes MAVS and thereby induces IFN production during viral infection	(52)
TBK1 and IKKβ	Phosphorylation	S442	Gives phosphorylated MAVS the ability to recruit IRF3 for its phosphorylation by TBK1	(53)
NLK	Phosphorylation	S121/S212/S258/S329	Inducing the degradation of MAVS in the later phase of infection, thereby inhibiting further signaling transduction	(54)
PPM1A/PP2Cα	Dephosphorylation	NA	Dephosphorylates MAVS, TBK1, and IKKε, thus blocking signaling conduction	(55)

#### Post-translational Positive Regulation of MAVS

Viral infection leads to K63-linked ubiquitination of MAVS at the mitochondrial outer membrane, which in turn induces MAVS aggregation, a marker of its activation. At present, ubiquitination by the E3 ubiquitin ligase TRIM31 is the only known initiator of MAVS aggregation and activation (Table 1). Notably, TRIM31-mediated MAVS aggregation does not occur under RIG-I deficient or non-viral infection conditions, thus it can be considered that RIG-I engagement is required for TRIM31-mediated MAVS aggregation after viral infection (39). MAVS glycosylation by O-linked N-acetylglucosamine (O-GlcNAc) transferase (OGT) enhances TRIM31-mediated K63-linked ubiquitination of MAVS (Table 1). Viral infection increases glucose metabolism in the host cell, including activation of the hexosamine biosynthesis pathway and elevation of OGT levels (40). Although only 2–5% of intracellular glucose enters the hexosamine biosynthesis pathway under physiological conditions, the ability of OGT to promote RLR signaling highlights the importance of glucose metabolism in antiviral innate immunity. Studies have shown that persistent MAVS aggregation may be associated with the occurrence of autoimmune diseases such as systemic lupus erythematosus. Further, the sustained expression of IFN and its regulatory genes and unsuppressed signals, will eventually result in tissue damages (56).

K27-linked ubiquitination by the E3 ubiquitin ligase TRIM21 also has a positive regulatory effect on MAVS. Viral infection upregulates the expression of TRIM21, thereby promoting the recruitment of TBK1 to MAVS and enhancing downstream signaling (Table 1) (41).

Phosphorylation is another post-translational modification that plays a key role in regulating MAVS signaling. After activation of TBK1 complexes by MAVS, the complex components TBK1 and IKK kinases phosphorylate MAVS, which then gains the ability to recruit IRF3. Recruited IRF3 is then phosphorylated by TBK1, which induces IRF3 homodimerization, translocation to the nucleus, and promotion of IFN transcription (Table 1) (53).

It is well-known that K48-linked ubiquitination of MAVS promotes its proteasomal degradation (44), and proteins that inhibit MAVS K48-linked ubiquitination can thus positively regulate RLR-mediated signaling. For example, the endogenous protein cyclophilin A, which normally competes with TRIM25 for MAVS binding, is upregulated by viral infection, thereby effectively inhibiting TRIM25-mediated MAVS ubiquitination and degradation (43, 57). Ubiquitination is a reversible process, and viral infection also upregulates the expression of ovarian tumor family deubiquitinase 4 (OTUD4), which removes K48-linked ubiquitin from MAVS and thus reduces its degradation (Table 1) (52). These findings suggest that the host cell has evolved mechanisms to inhibit K48-linked ubiquitination and degradation of MAVS during viral infection, thus ensuring that at least some level of signaling for the antiviral response is maintained.

Collectively, these studies have revealed that MAVS signaling is regulated via three main post-translational modifications that act in concert: (1) TRIM31-mediated K63-linked ubiquitination activates MAVS; (2) TRIM21-mediated K27-linked ubiquitination promotes the recruitment of TBK1 to MAVS; and (3) TBK1- and IKK-mediated MAVS phosphorylation promotes the recruitment of IRF3 to MAVS. In addition, inhibition of K48-linked ubiquitination and degradation is necessary to maintain an adequate cellular level of MAVS to ensure antiviral immunity.

#### Post-translational Negative Regulation of MAVS

At present, the negative post-translational regulation of MAVS is mainly manifested in K48-linked ubiquitination modification, signal blocking, autophagy, and apoptosis (Figure 3).

##### MAVS regulation by ubiquitination

As noted above, K48-linked ubiquitination of MAVS triggers its proteasomal degradation and thus limits RLR signaling. A number of E3 ubiquitin ligases have been shown to mediate MAVS K48-linked ubiquitination, including TRIM25, AIP4, Smurf1, Smurf2, MARCH5, and RNF5 (see also Table 1 for details). In addition, increasing evidence suggests that binding of various adaptor proteins to MAVS modulates MAVS ubiquitination-mediated degradation. For example, K48-linked ubiquitination of MAVS mediated by the E3 ubiquitin ligase AIP4 (also known as Itch) is promoted by the poly (C)-binding protein 2 (PCBP2), which is upregulated after viral infection. PCBP2 binds to the TM domain of MAVS and recruits AIP4, which promotes MAVS degradation (Table 1) (44). Recent work has established a similar role for the highly homologous PCBP1; however, PCBP1 expression level does not appear to be altered during viral infection (Table 1). Thus, it is possible that MAVS expression is maintained at a low level under physiological conditions in part by PCBP1-mediated promotion of degradation, whereas upregulation of PCBP2 during viral infection promotes MAVS degradation and acts as a negative feedback mechanism to re-establish homeostasis and avoid excessive immune signaling (45). AIP4 is also regulated by the adapter protein Tax1-binding protein 1 (TAX1BP1), which binds to the MAVS CARD and recruits AIP4. TAX1BP1 regulates MAVS degradation under physiological conditions and during viral infection in a similar manner to PCBP1/2 (Table 1) (46). The E3 ubiquitin ligase Smurf1 that mediates the K48-linked degradation of MAVS is known to be positively regulated by two adaptor proteins: OTUD1 and Nedd4 family-interacting protein 1 (Ndfip1). OTUD1 interacts with Smurf1 and functions as a deubiquitinase to remove the K48-linked ubiquitination of Smurf1, prevent its self-ubiquitination degradation, and upregulate its protein level; whereas the binding of Ndfip1 to MAVS recruits Smurf1 to MAVS, and the interaction between Ndfip1 and Smurf1 enhances the self-ubiquitination (possibly k63-linked) and enzyme activity of Smurf1 (Table 1) (47, 49). The diversity of E3 ubiquitin ligase involved in MAVS degradation suggests that they could provide more targets for the treatment of viral infections.

Several proteins negatively regulate antiviral innate immunity by inhibiting TRIM31-mediated K63-linked ubiquitination and activation of MAVS. One is Fas-associated factor 1 (FAF1), a protein that enhances Fas-induced apoptosis and was recently shown to be involved in the MAVS response. Under physiological conditions, the aggregates of FAF1 formed by its UBL domain, bind and antagonize MAVS by competing with TRIM31 for MAVS interaction, thereby inhibiting the MAVS aggregation. However, after viral infection, FAF1 is phosphorylated by activated IKKε, which promotes its depolymerization and lysosomal degradation and thus relieves MAVS inhibition (58). Rac1 is known to promote the growth of tumor cells. However, viral infection leads to geranylgeranylation and palmitoylation of Rac1, which inhibits the TRIM31–MAVS interaction and suppresses innate immunity; interestingly, under physiological conditions, Rac1 can also inhibit MAVS aggregation (59). It is known that TRAF3, an E3 ligase and located in the downstream signaling protein of MAVS, its self-ubiquitination of K63-linked promotes TRAF3-TBK1 interaction (60). However, K63-linked ubiquitination on Lys500 of MAVS (by a currently unidentified enzyme) can promote detachment of TRAF3 from MAVS, and trigger K63-linked deubiquitination on TRAF3 and its subsequent K48-linked ubiquitination degradation, thereby inhibiting TRAF3-mediated signaling (Table 1) (38).

Collectively, these studies suggest that MAVS K48-linked ubiquitination under physiological conditions serves to promote MAVS degradation and prevent spontaneous activation of the immune response. The same event occurring in the late stages of viral infection serves to restore cellular basal activity and prevent excessive production of potentially harmful immune mediators. Similarly, inhibition of TRIM31-mediated K63-linked ubiquitination of MAVS prevents its aggregation and activation, and also serves to maintain MAVS homeostasis.

##### MAVS blockade via direct protein interaction

Another mechanism by which MAVS signaling is held in check under physiological conditions is via direct binding of tubulin-tyrosine ligase-like protein 12 (TTLL12) to MAVS, TBK1, and IKKε (Figure 3). During viral infection, TTLL12 expression is reduced, thereby releasing the block in MAVS-mediated activation of the immune response (61). Protein phosphatase magnesium-dependent 1A (PPM1A, also known as PP2Cα) is a component of the TBK1/IKKε complex and normally maintains MAVS, TBK1, and IKKε in a dephosphorylated state, preventing MAVS downstream signaling (Table 1). However, upregulation of MAVS expression during viral infection overcomes the PPM1A-mediated block in signaling and serves as a threshold for activation of antiviral signaling (55).

Lactate, the end product of anaerobic glycolysis, has long been considered a metabolic waste product, but recent studies have shown that it has an important role in immunomodulation. Indeed, lactate acts as a key negative regulator of RLR signaling via direct binding to the MAVS TM domain, which prevents MAVS mitochondrial localization, aggregate formation, and signaling function. It is known that lactate level is directly related to glycolysis. Under physiological conditions, the interaction between hexokinase II (HK2), a key rate-limiting enzyme for glycolysis and located on the mitochondrial outer membrane, and MAVS maintains HK2 activity in its basal state, which ensures the operation of glycolysis metabolism and the suppression of MAVS mediated by lactate. However, during viral infection, binding of activated RIG-I to MAVS causes dissociation of HK2, leading to a reduction in glycolysis and lactate production, attenuation of lactate-mediated MAVS inhibition, and upregulation of downstream signaling for IFN production (62, 63). It reveals that the interaction of MAVS and HK2 is associated with its activation and function. Interestingly, downregulation of glucose metabolism and lactate-mediated repression of MAVS occurs only in the early stages of viral infection. This is consistent with the observation that viral infection is accompanied by an overall upregulation of glucose metabolism to ensure adequate energy supplies for replication and proliferation.

During the later stages of viral infection, MAVS activity is negatively regulated by UBX-domain-containing protein 1 (UBXN1), a member of the ubiquitin-binding protein family. The expression of UBXN1 is strongly upregulated late during viral infection and it competes with TRAF3/TRAF6 for binding to MAVS (amino acids 455–460), thus blocking MAVS signaling (64, 65). Gpatch domain-containing protein 3 (GPATCH3), a widely expressed protein, acts similarly to UBXN1. GPATCH3 binding to MAVS prevents the assembly of the MAVS/TRAF6/TBK1 complex during viral infection (66).

##### MAVS Regulation related to apoptosis and autophagy

When other mechanisms fail, apoptosis can be used as a strategy to inhibit viral replication. However, the cell has also evolved various feedback mechanisms to ensure timely inhibition of immune signaling to avoid excessive tissue damage. For example, NLRP11, a type I IFN-induced Nod-like receptor, is upregulated after viral infection and can inhibit type I IFN production. Recent studies demonstrated that part of NLRP11 translocates from cytoplasm to mitochondria and interacts with MAVS, but NLRP11 neither disrupts the interaction between MAVS and RIG-I nor the ubiquitination of MAVS. However, NLRP11 strongly interacts with TRAF6 and promotes K48-linked ubiquitination degradation of TRAF6, thereby inhibiting the RLR signaling. Interestingly, overexpression of NLRP11 inhibited the cleavage of poly-ADP-ribose polymerase (a molecule involved in apoptosis related to DNA damage) in cells while knockout of TRAF6 abrogated NLRP11-mediated inhibition (67). Because TRAF6 is involved in apoptosis after viral infection (68), NLRP11 can potentially inhibit apoptosis, which also ensures the survival of the host cells. In addition, the knockout of MAVS eliminated the interaction between NLRP11 and TRAF6 (67). These findings suggest that MAVS serves as a platform on which NLRP11 degrades TRAF6 and inhibits TRAF6-dependent apoptosis. The dual blocking role of NLRP11 in the antiviral immune response serves as a negative feedback mechanism for suppression of RLR signaling, IFN production, and apoptosis. Although cysteinyl aspartate specific proteinase-3 (caspase-3) is best known as a key executioner of apoptosis, its non-apoptotic functions are attracting increasing attention. During viral infection, activated caspase-3 cleaves MAVS at Asp429 and Asp490 and inhibits excessive signaling (69). This observation expands our understanding of the functions of caspase-3 and provides new insights for the development of drugs to prevent aberrant immune responses. In addition, the hemagglutinating virus of Japan envelope (HVJ-E) derived from inactivated replication-defective Sendai virus was found to have antitumor activity dependent on RIG-I/MAVS signaling. HVJ-E activates the RIG-I/MAVS signaling to upregulate the expression of its downstream pro-apoptotic genes TRAIL and Noxa, and thus inducing selective apoptosis of tumor cells (including prostate cancer cells, lung cancer cells, and breast cancer cells). Remarkably, this process does not occur in normal immune cells (70, 71).

Autophagy, a cellular degradation process, also acts as an antiviral defense mechanism by clearing intracellular microorganisms and interacting with the innate immune response. For example, Tetherin (also known as BST2 or CD317) is an IFN-induced membrane protein that is upregulated following viral infection. Tetherin induces K27-linked ubiquitination of MAVS by recruiting the E3 ubiquitin ligase MARCH8. The autophagy protein NDP52 then binds to K27-linked ubiquitinated MAVS, leading to lysosomal degradation. Tetherin-mediated autophagic degradation of MAVS can thus be considered another negative feedback mechanism that suppresses excessive signaling (42).

Additional negative regulatory mechanisms in MAVS-mediated antiviral immunity include phosphorylation of MAVS by Nemo-like kinase, which occurs in the later stage of viral infection and induces MAVS degradation (54). The mitochondrial fusion proteins Mfn1 and Mfn2, located on the mitochondrial outer membrane, play opposing roles in targeting MAVS during RLR-induced signaling. Mfn2 interacts with the C-terminal (particularly TM domain) of MAVS through a HR1 region and thus blocking the activation of IRF3 and NF-κB, and the process is based on an Mfn2-dependent complex (~600 kDa). The knockdown of endogenous Mnf2 reduced the relative molecular mass of MAVS complex from high molecular mass to a lower molecular mass and enhanced the antiviral effect of MAVS (72). However, there is evidence that the MAVS aggregate is larger than 700 kDa (28), so speculating that the MAVS complex may be composed of MAVS and its downstream signaling molecules, and this Mfn2-dependent form is not conducive to signaling transmission. In contrast to Mfn2, Mfn1 plays a positive role by regulating the mitochondrial redistribution of MAVS to form its speckled staining pattern in cells, which might relate to the formation of MAVS prion-like structures (73). Collectively, homologous proteins Mfn1 and Mfn2 fine-tune the MAVS-mediated signaling, in addition to ensuring the regulation of mitochondrial fusion. Furthermore, Mfn1/Mfn2-dependent mitochondrial fusion can enhance RLR signaling (74), the specific mechanism remains unclear.

## Regulation of MAVS by viral proteins

Viruses can escape the host antiviral immune response by promoting the cleavage or degradation of MAVS and by directly interfering with RLR-activated signaling pathway components. Notably, there is little evidence that viral proteins play a role in enhancing the host innate immune response.

### Cleavage of MAVS

**Figure 4:**
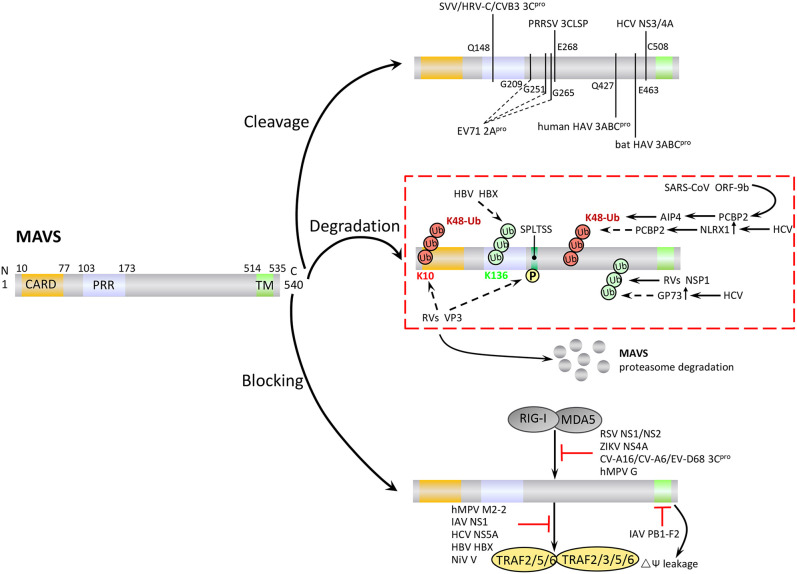
Negative regulation of MAVS signaling by viral proteins. Viruses employ various strategies to escape the host antiviral immune response, including cleavage or proteasomal degradation of MAVS, and direct binding to signaling molecules to block the RLR pathways.

Many virus-encoded proteins are proteases and can cleave MAVS independently of proteasomal degradation or apoptosis to inhibit RLR signaling (Figure 4). The first viral protein reported to colocalize with and cleave MAVS at the mitochondria was hepatitis C virus (HCV) serine protease NS3/4A. NS3/4A cleaves MAVS at Cys508, which dislodges the N-terminal fragment of MAVS from the mitochondria, reduces downstream signaling, and enables persistent viral infection (75). The small RNA viruses Seneca Valley virus, human rhinovirus C, and coxsackievirus B3 (CVB3) all encode a cysteine protease, 3C^pro^, which cleaves MAVS at Gln148 and inhibits its activity (76–78). CVB3 also produces a second MAVS-cleaving protease, 2A^pro^, although the specific cleavage site is unclear (79). In addition, a 3C-like serine protease (3CLSP), which is similar to 3C^pro^, is produced by porcine reproductive and respiratory syndrome virus and cleaves MAVS at Glu268 (80). The viral-encoded proteases 3ABC (produced by hepatitis A virus, HAV) and 2A^pro^ (enterovirus 71, EV71) are particularly noteworthy. 3ABC produced by bat and human HAV cleave human MAVS at Glu463 and Gln427, respectively, highlighting the possibility that cross-species interference with MAVS signaling may promote the transfer of HAV between species (81). The first viral protein reported to cleave MAVS at multiple amino acid residues was the cysteine protease 2A^pro^, which is produced by EV71, the main cause of hand, foot and mouth disease. 2A^pro^ cleaves MAVS at Gly209, Gly251, and Gly265 (82).

### Degradation of MAVS

In addition to the direct cleavage, some viral proteins induce proteasomal degradation of MAVS (Figure 4). For example, the hepatitis B virus (HBV) protein X (HBX) binds to MAVS and promotes its ubiquitination at Lys136, leading to proteasomal degradation. However, it is unclear which E3 ligase is involved in this process (83). Open reading frame 9b (ORF-9b), a protein encoded by coronavirus SARS, catalyzes the K48-linked ubiquitination and degradation of the MAVS signalosome (MAVS, TRAF3, and TRAF6) via the PCBP2–AIP4 axis. The degradation of mitochondrial fission protein Drp1 triggered by ORF-9b can reduce IFN signaling, suggesting that ORF-9b may also contributes to viral escape by manipulating mitochondrial function (84). Rotaviruses (RVs) use multiple mechanisms to degrade RLR pathway signaling components. VP3, an RV structural protein, induces phosphorylation of the newly discovered MAVS motif SPLTSS (residues 188–193), leading to K48-linked ubiquitination and proteasomal degradation of MAVS (85). Interestingly, the NSP1 protein of RVs has E3 ubiquitin ligase-like activity and can target proteins for ubiquitination (86). For example, NSP1 can bind to MVAS CARD or TM domain to promote ubiquitin-dependent proteasomal degradation (87). NSP1 also induces ubiquitin-proteasomal degradation of IRF3/5/7 and the NF-κB activating factor β-TrCP, and induces degradation of RIG-I in a proteasome-independent manner (88–90).

HCV infection upregulates the expression of Golgi protein 73 (GP73), a serum marker of liver disease and hepatocellular carcinoma, and promotes the coiled-coil domain of TRAF6 to recruit GP73 to MAVS. GP73 binding leads to the degradation of MAVS and TRAF6 through a proteasome-dependent pathway, thereby supporting HCV infection (91). HCV infection also upregulates NLRX1 (also known as NOD5/NOD9/NOD26) and induces MAVS K48-linked ubiquitination and degradation via PCBP2. Neither NLRX1 nor PCBP2 has E3 ubiquitin ligase activity, and it seems likely that PCBP2 may recruit AIP4 to mediate the ubiquitination step (92).

### Modulation of MAVS Signaling

Viral proteins can block MAVS-mediated signaling by direct or indirect inhibition of individual pathway components (Figure 4). For example, a number of viral proteins bind to and block the interaction between MDA5, RIG-I, and MAVS. These include NS1 and NS2 of respiratory syncytial virus, NS4A of Zika virus, and 3C^pro^ proteins of coxsackievirus (CV)-A16, CV-A6, and EV-D68 (93–96). Human cytomegalovirus-encoded glycoprotein US9 disrupts mitochondrial integrity and induces loss of membrane potential, which leads to detachment of MAVS (97). The virulence factors M2-2 and glycoprotein G of human metapneumovirus (hMPV) both block RLR signaling. M2-2 binds to MAVS, blocking formation of MAVS/TRAF signalosomes, while glycoprotein G binds to the CARD of RIG-I, thus blocking MAVS binding (98, 99). The capsid protein VP16 of herpes simplex virus 1 blocks the production of early ISGs mediated by peroxisome MAVS, inhibiting the early peroxisome-mediated response to viral infection (100).

Influenza A virus (IAV), the most common type of influenza virus, has caused many worldwide pandemics because of its variability and high pathogenicity. The H5N1 strain of IAV encodes PB1-F2 protein and PB2Δ, a defective but functional polypeptide, which play opposing roles in MAVS-mediated antiviral innate immunity. PB1-F2 binding to the MAVS TM domain reduces the mitochondrial membrane potential and disrupts MAVS signaling, whereas PB2Δ directly interacts with MAVS to promote signaling for type I IFN production, thereby inhibiting virus replication (Figure 4) (101, 102). IAV NS1 inhibits TRAF3–MAVS interactions by removing K63-linked ubiquitination on TRAF3, negatively regulating the antiviral response (103). IAV also inhibits MAVS expression at the post-transcriptional level. For example, IAV upregulates the expression of miRNA-125a and miRNA-125b, which bind to the MAVS mRNA 3'UTR and inhibit its translation (104). In addition, the M2 protein of IAV induces Ca^2+^-dependent reactive oxygen species production, which enhances MAVS aggregation and activity (105).

Viral proteins can also promote intracellular proteins to inhibit MAVS activity (Figure 4). For example, HCV NS5A promotes the mitochondrial protein leucine-rich PRR motif-containing protein (LRPPRC) binding of MAVS to block the association of MAVS and TRAF3/6 (106). HBX can promote recruitment of the E3 ubiquitin ligase linear ubiquitin chain assembly complex (LUBAC) to the mitochondria, and the subsequent linear ubiquitination of MAVS blocks MAVS–TRAF3 interactions (107). The V protein of Nipah virus inhibits degradation of the negative regulatory factor UBXN1, thereby enhancing blockade of MAVS–TRAF interactions by UBXN1 (65). Several viruses employ multiple strategies to antagonize antiviral immunity. For example, HCV not only cleaves MAVS via N3/4A but also induces the proteasomal degradation of MAVS via GP73 or NLRX1.

Although most current studies focus on hepatitis viruses (HAV, HBV, and HCV) and respiratory viruses (IAV, human rhinovirus C, SRAS coronavirus, respiratory syncytial virus, EV-D68, and hMPV), increasing attention is being paid to viruses such as EV71, CV-A16, CV-A6, Nipah virus, Seneca Valley virus, and Zika virus. This work will undoubtedly add to our understanding of the mechanisms by which viruses evade the host antiviral response.

## Conclusion

Recognition that MAVS plays a pivotal role in antiviral immunity has led to a surge in research on this molecule. It is now clear that cells and viruses regulate MAVS at both the post-transcriptional and post-translational levels using multiple mechanisms. Among them, ubiquitination plays a particularly crucial role in initiating, maintaining, and curtailing RLR-mediated signaling pathways, with K27-, K48-, and K63-linked ubiquitination acting in concert to regulate MAVS signaling. Whether ubiquitination of other types (e.g., K6-, K11-, K29-, K33-, and M1-linked ubiquitin chains) can affect the biological functions of MAVS is unclear. The manner in which MAVS is affected by other modifications, such as phosphorylation and glycosylation as well as products of anaerobic glycolysis (lactate) and components of the apoptosis and autophagy pathways are attracting increasing interest. Viral replication requires a considerable uptake of nutrients from the host organism, and whether other components of carbohydrate and protein metabolism can regulate the antiviral immune response is another new field of study. As the key adaptor protein of RLR-mediated signaling, MAVS clearly plays a crucial role in the innate immune response. Despite significant advances in recent years, as illustrated in this review, the mechanisms regulating MAVS-mediated signaling in other species remains unclear. Further work in this area is expected to provide a clearer understanding of MAVS activity in antiviral immunity and to fuel the discovery of new drugs for the treatment of viral infection.

## Author Contributions

ZR carried out literature search and designed idea. TD sorted out important background information and drafted the manuscript. ZZ and ZX critically revises the content of the manuscript. JD and ZW performed the manuscript review. All authors read and approved the final manuscript.

## Conflict of Interest

The authors declare that the research was conducted in the absence of any commercial or financial relationships that could be construed as a potential conflict of interest.
